# Effects of a structured education program on glycemic control in type 1 diabetes

**DOI:** 10.1590/2359-3997000000278

**Published:** 2017-06-23

**Authors:** Ana Paula F. Pacheco, Simone van de Sande-Lee, Rita de Cássia B. Sandoval, Sônia Batista, Jefferson L. B. Marques

**Affiliations:** 1 Centro de Ciências da Saúde Universidade Federal de Santa Catarina Florianópolis SC Brasil Programa de Pós-Graduação em Ciências Médicas, Centro de Ciências da Saúde, Universidade Federal de Santa Catarina (UFSC), Florianópolis, SC, Brasil; 2 Hospital Universitário UFSC Florianópolis SC Brasil Hospital Universitário, UFSC, Florianópolis, SC, Brasil; 3 Instituto de Engenharia Biomédica Departamento de Engenharia Elétrica e Eletrônica UFSC Florianópolis SC Brasil Instituto de Engenharia Biomédica, Departamento de Engenharia Elétrica e Eletrônica, UFSC, Campus Universitário – Trindade, Florianópolis, SC, Brasil

**Keywords:** Diabetes mellitus, type 1, education, self-care, quality of life

## Abstract

**Objective:**

Diabetes mellitus is associated with significant morbidity and mortality, and education is known to play a key role in managing this disease. This study addresses the effects of a structured education program (SEP) on self-care in subjects with type 1 diabetes mellitus (T1DM). The aim was to evaluate the effect of a SEP on glycemic control, knowledge, and skills associated with diabetes care in subjects with T1DM.

**Subjects and methods:**

A total of 47 adults with T1DM were followed up for 20 months (32 participated in the SEP and 15 served as a control group). The SEP consisted of workshops, individualized care, 24-hour distant support, and a questionnaire assessing knowledge of diabetes care. Glycosylated hemoglobin (HbA1c) levels were measured before and after the SEP implementation.

**Results:**

Compared with pre-SEP levels, the mean HbA1c levels decreased by approximately 20% (21 mmol/mol) at 1 year, with a further 11% reduction (10 mmol/mol) observed 8 months later (p < 0.001). Knowledge about diabetes care increased by 37% between the pre-SEP and post-SEP questionnaires (p < 0.005).

**Conclusion:**

Relevant improvements occurred after SEP activities. The sustained decrease in HbA1c levels and the overall increase in knowledge and confidence regarding diabetes care reinforce the importance, necessity, and positive outcomes of a SEP intervention in T1DM.

## INTRODUCTION

Diabetes mellitus (DM), one of the diseases with the highest mortality rates, is considered a worldwide epidemic (1). According to the International Diabetes Federation (IDF) 2015 Update, DM affects 15.8% of the population aged 20-79 years in Brazil, and has been associated with approximately 130,700 deaths. DM is also the leading cause of hospitalization for complications such as cardiovascular disease, dialysis for chronic renal failure, and lower limb amputations (2). The number of cases of DM worldwide reached 415 million in 2015, of which 5-10% were type 1 DM (T1DM).

Although T1DM affects only a minority of the patients with DM, it is responsible for a great share of the serious complications of the disease. T1DM is usually diagnosed in youths (3) and requires continuous measures such as diet, medication, and lifestyle changes (2). Less than 1 in 5 individuals with DM achieves the recommended glycemic targets, which increases the risk of development of chronic complications (4). With an increasing public health burden arising from chronic diseases such as DM, emphasis is placed on the patient to take responsibility for managing his own disease.

Patient education can lead to improved knowledge and self-management skills (5), reducing the risks of complications (6). However, qualitative studies based on the patients’ own understanding and views regarding T1DM have shown concern about these patients’ current information needs (7,8), including administration of medication, management of hypoglycemia, glucose testing, diet, following of sick-day guidelines, foot care, and understanding of HbA1c results (9-12). In Germany, an approach of structured education in health has been delivered to thousands of individuals with T1DM and successfully introduced in an outpatient setting (11,13). Other countries should also provide health services and DM education tailored to the patients’ individual, social, and cultural needs (14-16).

An exemplary education approach offered to T1DM patients in the United Kingdom is the DAFNE (Dose Adjustment For Normal Eating) program (14). The program includes courses with topics on physical activity, nutrition, management of hypoglycemia, alcohol consumption, sickness, potential complications, and pregnancy (15). While it has been recognized that the skills of DM self-management are best provided by structured education programs (SEPs) delivered by professionals with appropriate training (14), more needs to be known about patient self-care behavior and the perceived barriers which influence an individual’s decision to effectively managing his DM. Additionally, offering DM education in groups may also be very effective (16). If we are to improve the effectiveness of educational interventions, we ought to record the patients’ characteristics and psychosocial variables and explore with details the difficulties and barriers they face to implement and sustain DM self-management (17,18). Reports have shown that patients entering DAFNE with an HbA1c > 8.5% (69 mmol/mol) experienced a 0.8% (9 mmol/mol) decrease in HbA1c levels at 1 year, with a subsequent health economic analysis concluding that the intervention was highly cost-effective and would pay for itself within 3 years (17).

Based on the considerations above, the objectives of this study were to 1) evaluate the effect of a SEP on HbA1c levels in individuals with T1DM; 2) assess the acquisition of knowledge by the participants, identifying the most challenging areas; and 3) elaborate an approach to structured education in health based on DAFNE, along with a self-care incentive for individuals with T1DM, providing these individuals with means to improve their knowledge about the disease and self-care management skills.

## SUBJECTS AND METHODS

### Study design and ethics

This was a prospective, cohort study approved by the Ethics Committee on Human Research at Federal University of Santa Catarina.

### Subjects

We included 47 consecutive subjects with T1DM, of both genders, and attending the Endocrinology Clinic at the University Hospital in Florianopolis, who met the following inclusion criteria: clinical diagnosis of T1DM for at least 5 years, minimum age of 18 years, glycosylated hemoglobin (HbA1c) > 7% (53 mmol/mol), and literacy. The exclusion criteria comprised refusal to participate in the study, disorder or disability preventing attendance at the activities, and residence outside the Florianopolis metropolitan area. All subjects were invited to participate in the SEP; 32 of them accepted and comprised the SEP group, while the remaining 15, who were unable or unwilling to participate in the SEP, constituted the control group.

### Pre-SEP and post-SEP evaluations

All subjects underwent clinical examination and blood collection for measurement of HbA1c levels at three time points: baseline (pre-SEP), after the intervention (post-SEP; approximately 1 year after the beginning of the study); and at the end of the study period at 20 months (follow-up). Levels of HbA1c were determined at the University Hospital laboratory using the ion exchange chromatography method.

The questionnaire used by the DAFNE program was applied to evaluate the previous knowledge of the participants about DM care. The questionnaire was translated into Portuguese, adapted to the Brazilian population by the investigators, and applied to 30 subjects (25 in the SEP group and 5 in the control group) at two time points, pre-SEP and post-SEP. The questionnaire comprises 22 questions scoring between 0-10 and totaling 220 maximum points, and is divided into four categories: 1) food/carbohydrates and physical activity, 2) insulin regimen, 3) management of blood glucose, and 4) complications.

### Structured education program

The program consisted of group meetings (workshops) during the first 6 months, which were planned and carried out in a multidisciplinary scenario according to the topics to be discussed. A total of 32 individuals participated in this stage, which equated to approximately 10 individuals per session, with each individual attending three group meetings. Corresponding to DAFNE guidelines, they also attended individual consultations with the nurse and the nutritionist, which occurred once a week or according to the patients’ needs during the SEP period, with psychological referral being offered when necessary. The communication with these individuals occurred via landline and mobile phones, e-mail, social networking, and intelligent web system.

The 15 individuals who comprised the control group attended routinely the outpatient clinic, but did not participate in any of the SEP activities. For the SEP group, we discussed each individual’s daily activities, carbohydrate count, information regarding healthy eating, and blood glucose levels. We also discussed the participants’ insulin management, insulin application with syringe/pen, selection of the best insulin injection site and the reasons for that, how to react when hypoglycemia occurs, information regarding hyperglycemia and ketoacidosis, how to proceed at parties/events, ingestion of alcohol, consumption of different types of food, changes in daily routine, how to perform treatment in case of pregnancy, how to act when travelling and/or living in a different environment than the usual one, and finally, the most appropriate actions when practicing light, moderate, and strenuous exercise.

A differential methodology was implemented under an “immediate” clinical support, in which the subjects had a direct contact with a full-time nurse for clarification of urgent matters, so they would not need to wait for the next appointment or meeting to resolve their concerns. The means of contact used for this purpose depended on the need or urgency. In this case, the contact was reciprocal: the participants contacted us when necessary, and we contacted them to monitor and assess the achievement of the goals proposed in the consultation.

### Semistructured interview

At the end of the study period (20 months), the subjects in both the SEP and control groups underwent a qualitative semistructured interview with the aim of obtaining their feedback about the methodology applied. This interview included 10 questions with four possible answers: (1) a lot, (2) moderate, (3) little, and (4) no/nothing/none. These questions evaluated the participant’s self-confidence, assessment of the clarifications received during the program or by the staff of the clinic, satisfaction in self-care, satisfaction with quality of life, diet flexibility, assessment of the questions posed by the support staff at the outpatient clinic, motivation, acceptance of the condition of having DM, and interest in continuing the treatment approach they had just received (SEP).

### Statistical analysis

The results of the clinical data are expressed as mean absolute values (± standard deviation [SD]) as well as percentages, when appropriate. A p value < 0.05 was considered statistically significant.

#### Questionnaire

Statistical differences between the parameters obtained in the pre-SEP and post-SEP time points were determined using Student’s paired t test, and differences between parameters in the SEP group and control group were assessed with Student’s independent samples t test.

#### Glycosylated hemoglobin and body mass index

Statistical differences in HbA1c levels between the pre-SEP, post-SEP, and follow-up time points were determined by repeated measures analysis of variance (ANOVA), while those related to body mass index (BMI) values between the SEP and control groups were determined with Student’s independent samples t test.

## Semistructured interview

The data related to the semistructure interview were described and analyzed in an Excel spreadsheet, and the results were generated in graphical form.

## RESULTS

A total of 47 subjects with T1DM were selected for this study, including 25 (53%) females and 22 (47%) males. All participants had a diagnosis of T1DM for at least 5 years and a maximum of 31 years, with a mean of 12.9 ± 6.8 years. In terms of age, they ranged between 18 and 44 years, with a mean of 26 ± 6.7 years among males and 28 ± 8.7 years among females.

Overall, 32 (68%) patients participated in the SEP and 15 (32%) comprised the control group. The main reasons for patients declining to attend the program were residence far from the hospital or incompatibility with work schedule. Among the participants in the SEP group, 12 (37.5%) were male, and 20 (62.5%) were female, and among those in the control group, 10 (67%) were male, and 5 (33%) were female. The baseline characteristics of the groups are depicted in Table 1.

**Table 1:** Baseline characteristics of the subjects

	Control group			SEP group		
	
	M	F	Total	M	F	Total
n	10	5	15	12	20	32
Age (yrs)	26 ± 6.9	32 ± 10.4	28 ± 8.4	26 ± 6.8	27 ± 8.2	26.5 ± 7.6
Diabetes duration (min-max) (yrs)	5-23	6-31	5-31	5-23	5-26	5-26
Weight (kg)	70 ± 9.5	61 ± 6.6	66 ± 9.3	77 ± 12.2	70 ± 12.2	73 ± 11.8
Height (m)	1.73 ± 0.04	1.56 ± 0.07	1.66 ± 0.1	1.75 ± 0.07	1.65 ± 0.05	1.69 ± 0.07
BMI (kg/m^2^)	23.4 ± 2.6	25.3 ± 4.1	24.1 ± 3.2	25.3 ± 3.0	26.1 ± 3.9	25.8 ± 3.5
Fasting glucose (mg/dL)	211 ± 79	177 ± 67	198 ± 75	190 ± 47	165 ± 62	175 ± 57
HbA1c (%)	10.7 ± 1.7	9.6 ± 1.7	10.4 ± 1.8	10.4 ± 1.9	10.2 ± 2.0	10.3 ± 1.9

Data are shown as mean ± standard deviation (SD), except for diabetes duration, which is shown as the minimum and maximum number of years since diagnosis. M: male, F: female, BMI: body mass index.

One patient in the SEP group died from complications of systemic lupus erythematosus and was excluded from the analysis. Otherwise, no hospitalizations or serious complications were reported during the study period. All DM supplies were provided by the publicly funded Brazilian health care system (SUS). However, due to a temporary problem with the supply of glucose test strips during the study, the strips were provided to the patients by the investigators during this time.

### Questionnaire

At baseline (pre-SEP), the percentage of correct answers were as follows: insulin regimen, 47 ± 3.1%; eating and carbohydrate counting/physical activity, 35 ± 3.2%; management of glucose levels, 65 ± 1.8%; complications, 40 ± 3.7%; and total hits, 48 ± 1.9%.

At post-SEP, the corresponding results were: insulin regimen, 87 ± 2.5%; eating and carbohydrate counting/physical activity, 83.5 ± 2.6%; blood glucose levels management, 91 ± 1.9%; complications 80 ± 2.2%; and total hits, 86 ± 1.4%.

**Figure 1:**
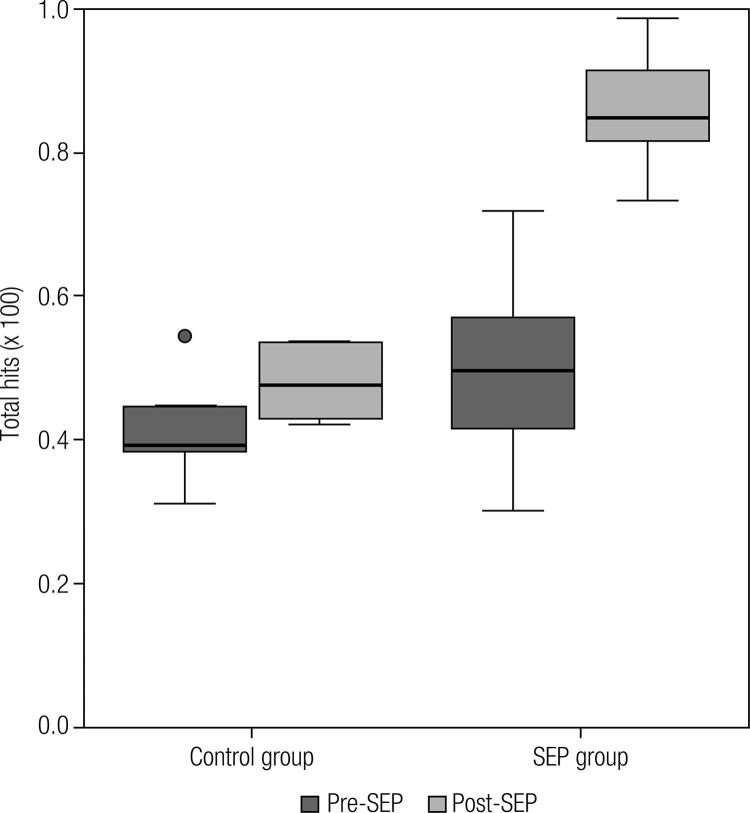
Comparison of the total hits on the questionnaire about diabetes care knowledge, in the pre-SEP and post-SEP evaluations.

There was a significant difference (p < 0.05) between pre-SEP and post-SEP results among the subjects who participated in the SEP program (of note, the mean scores for “total hits” increased by 37% from 49.5 ± 1.0% to 86 ± 0.6%). In contrast, the difference between the two time points was not significant in the control group (from 41.5 ± 0.9% to 48 ± 0.5%, respectively) (Figure 1).

### Glycosylated hemoglobin and body mass index

**Figure 2:**
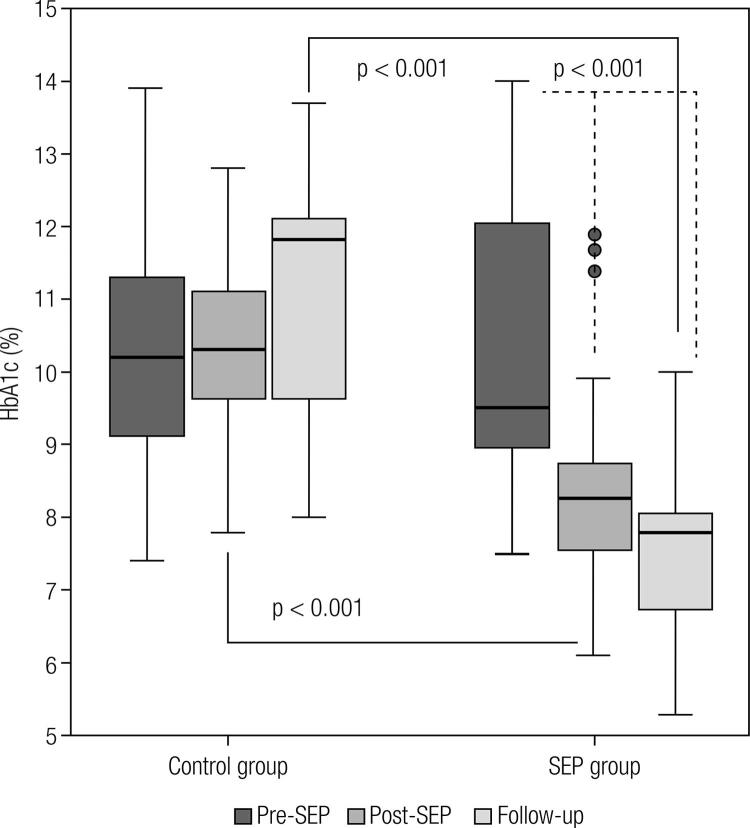
Glycosylated hemoglobin (HbA1c) values of 47 subjects (32 in the SEP group and 15 in the control group) at three time points (pre-SEP, post-SEP, and follow-up).

At pre-SEP, the mean HbA1c levels were 10.7 ± 1.7% (93 ± 19 mmol/mol) in males and 9.6 ± 1.7% (81 ± 19 mmol/mol) in females in the control group, and 10.4 ± 1.9% (90 ± 21 mmol/mol) in males and 10.2 ± 2.0% (88 ± 22 mmol/mol) in females in the SEP group. The corresponding levels at post-SEP were 10.5 ± 1.4% (91 ± 15 mmol/mol) in males and 10.2 ± 1.5% (88 ± 16 mmol/mol) in females in the control group, and 8.5 ± 1.4% (69 ± 15 mmol/mol) in males and 8.4 ± 1.4% (68 ± 15 mmol/mol) in females in the SEP group, and at the end of the study (follow-up), they were 11.0 ± 1.7% (97 ± 19 mmol/mol) in males and 11.1 ± 1.8% (98 ± 20 mmol/mol) in females in the control group compared with 7.5 ± 1.0% (58 ± 11 mmol/mol) in males and 7.5 ± 1.2% (58 ± 13 mmol/mol) in females in the SEP group. Repeated measures ANOVA using Greenhouse-Geisser correction showed a significant effect of time on HbA1c levels recorded at different stages of the program (pre-SEP, post-SEP, and follow-up; p < 0.001). In addition, there was a significant interaction (p < 0.001) between groups (control and SEP groups) and time. Similarly, pairwise comparisons between the control and SEP groups showed a significant difference (p < 0.001). These results are shown in Figure 2.

The mean baseline and final BMI values were 24.1 ± 3.2 kg/m^2^ and 23.8 ± 3.3 kg/m^2^, respectively, in the control group and 25.8 ± 3.5 kg/m^2^ and 25.6 ± 3.6 kg/m^2^, respectively, in the SEP group (all non-significant, p > 0.05).

### Qualitative semistructured interview

Overall, there was growing improvement in the health status of the participants in the SEP group, which led to improved general physical and mental statuses and reflected on their quality of life. In order to obtain feedback on the program, we sought the opinion of both participants and non-participants (SEP and control groups, respectively) using semistructured interviews on some important points.

**Figure 3:**
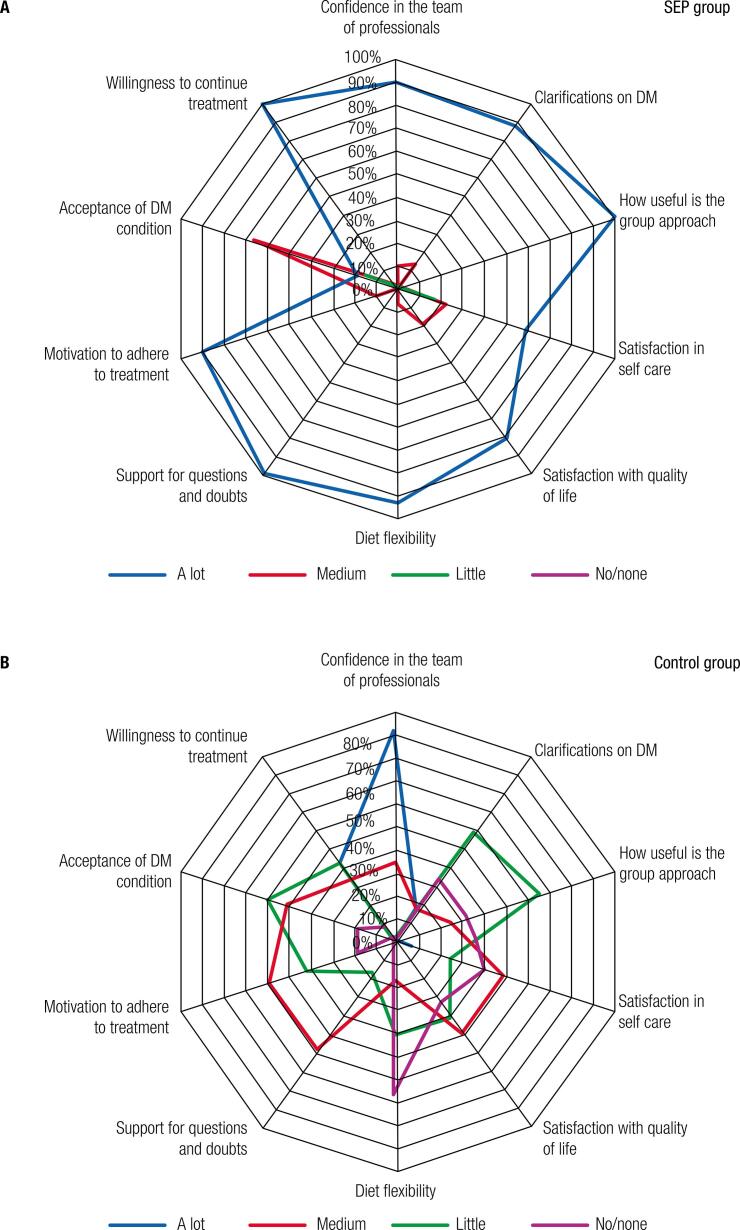
Patients’ feedback about the program: results of a semistructured interview with subjects from the SEP group (A) and the control group (B).

Among the individuals who participated in the SEP, 91% reported having developed a lot of confidence in the health team, 87.5% received substantial clarification about T1DM, 100% found the meetings in the T1DM group to be very useful, and 59% were very satisfied with their self-care relative to the T1DM (compared with 22% who were moderately satisfied and 19% who were somewhat satisfied). Furthermore, 81% of the participants were very satisfied with their quality of life, 94% were very satisfied with the flexibility of their diet, 100% were very satisfied with the questions posed by the support team, 91% were very motivated to continue with their treatments, and 19% were very accepting of their condition of having T1DM (versus 66% who were moderately accepting and 16% who just accepted this condition). Lastly, 100% said they would like to continue participating in the SEP if a group of T1DM patients was formed. These results are shown in Figure 3A.

Among the individuals in the control group, 73% reported having a lot of confidence in the health team (while 27% had moderate confidence); 47% received some clarifications about T1DM (but 26% answered no/none, and 13% answered a lot). In this group, 0% said that they thought the T1DM group was very useful (while 20% felt it was moderately useful and 53% and 27% found little or did not find anything useful, respectively). Furthermore, 33% reported having no satisfaction in their self-care in relation to the T1DM (versus 20% who had low satisfaction, 40% moderate, and 7% a lot). Regarding satisfaction in quality of life, 0% answered having a lot (while 40% had moderate, and 27% believed they had little). In terms of diet flexibility, 0% reported having a lot of flexibility (versus 33% who reported poor flexibility and 53% reported having no flexibility). None of the patients (0%) in the control group reported having a lot of support for questions (versus 46% who reported having moderate support). Concerning motivation for treatment, 7% reported having a lot (while 33% said they had little). Moreover, 0% said they were entirely accepting of their condition of having T1DM (while 40% moderately agreed, 46% somewhat agreed, and 13% did not agree). Lastly, 7% said they did not wish to participate if a group with T1DM was formed. These results are shown in Figure 3B.

## DISCUSSION

The results of this study show that individuals with T1DM have a deficit of information/knowledge about this condition, which coupled with the continued lack of professional support for self-care, prevents them from achieving effective self-management. Therefore, we observed significant improvements in knowledge and glycemic control after SEP activities.

In the original DAFNE trial, the mean HbA1c levels decreased from 9.4% (79 mmol/mol) to 8.4% (68 mmol/mol) 6 months after the training and increased to 8.9% (74 mmol/mol) at 12 months but remained significantly improved compared with baseline (17). A follow-up study of the participants in the original trial showed that after 4 years they still maintained a significant HbA1c improvement of 0.36% (4 mmol/mol) from baseline levels (13). Even 7 years after training, a persistent and clinically relevant reduction of 0.3% (3 mmol/mol) in HbA1c level remained (19).

More recent studies using DAFNE-based interventions have shown that this method is associated with modest improvements in glycemic control. The benefits of the method in routine clinical practice were assessed 1 year after the intervention, compared with baseline assessment, in 639 participants in the United Kingdom. The mean HbA1c levels decreased from 8.51% to 8.24%, while the participants experienced a greater sense of well-being and less psychological distress (20). Similarly, an evaluation of the outcomes of 145 participants undergoing DAFNE training at Australian DM centers showed improved quality of life and an HbA1c decrease from 8.2% to 7.8% at 1 year (21). The differences in the magnitude of HbA1c decrease observed in the above-mentioned studies when compared with the initial DAFNE trial were most likely related to different baseline values in both studies, as a greater decrease is expected with poorer baseline glycemic control. Moreover, the insulin regimen may have varied according to the time when each study was conducted.

In the present study, the mean HbA1c levels decreased by 1.9% (21 mmol/mol) at 1 year and further by 0.9% (10 mmol/mol) over the following 8 months, showing a significant 2.8% decrease (31 mmol/mol). Additionally, the participants in the present study presented lower HbA1c values after the intervention than those in the first DAFNE trial cohort (17). One reason for this large variation is clearly our participants’ exceedingly high baseline HbA1c values. In addition, both studies employed different approaches: while the original DAFNE course involved a 5-day outpatient program for a group of 6-8 people, we offered a DAFNE-based SEP with group meetings, individual consultations, and remote access on demand. We believe that this model of assistance accounted at least partially for the differences observed regarding HbA1c improvement, and we recommend that such approaches should be considered in future education programs planning. Although the impact of a single DAFNE course on glycemic control in the long term has been demonstrated, further interventions are required to help patients achieve the recommended HbA1c targets.

The main limitation of our study was the difference in HbA1c levels observed between the SEP and control groups, which may be partially due to a selection bias, as subjects in the present study were not randomized but, rather, selected through convenience sampling. Patients who choose to attend an education program are more likely to adhere to the proposed treatment and, therefore, show more improvements. Nevertheless, the highly significant intragroup differences between time points indicate that the SEP was an effective intervention in terms of glucose control. An additional limitation was the lack of data about insulin doses at the beginning and end of the study. However, all individuals were followed up by the same medical team who performed the dose adjustments according to the patients’ glycemic control during medical consultations, regardless of them participating or not in the SEP. Therefore, we believe that the program was the main factor that led to the HbA1c improvement. Finally, a previously validated translation of the questionnaire was not available; therefore, comparisons with international studies would not be reliable. The transcultural adaptation and validation of the DAFNE questionnaire into Portuguese would strengthen the results of studies on DM education programs in our country and should be performed as a next step.

In the interview feedback, the analysis showed that the SEP participants improved their knowledge about T1DM and self-confidence to carry out their treatment, demonstrated increasing satisfaction with diet flexibility, improved their quality of life, and were motivated to continue controlling and taking care of their health. The only topic that failed to show a positive response greater than 50% was the full acceptance of their T1DM (19%), which was moderately accepted by 65% of the respondents. This may be considered a natural behavior since even individuals who comply with their treatments are not necessarily comfortable with the idea of having a chronic disease.

Despite the success and influence of the SEP initiative, much work still needs to be done. A Brazilian survey including 6,671 adults with DM showed that 90% of the patients with T1DM were poorly controlled, and participation in a DM health education program was one of the characteristics significantly associated with improved glycemic control (22). Education in DM should no longer be regarded as an extra option in the treatment of the disease: it should be considered as essential as medication and, therefore, resourced, researched, evaluated, and quality assured to a similar standard. This study demonstrated that when gaps in the knowledge of self-care and treatment are identified in individuals with T1DM, these individuals can receive better information about their disease and its possible consequences, therefore empowering them to manage this chronic health condition.

The SEP based on DAFNE showed significant results achieving the original objectives. The participants reached a higher degree of information and knowledge about DM, improved their skills in daily self-care (as demonstrated by the results of the questionnaires and interviews), and reported in the consultations and workshops more confidence to perform the correct treatment. Furthermore, the decrease in HbA1c levels among the subjects who participated in the SEP was significant when different time points (pre-SEP and post-SEP) were compared, and a further decline was observed at the end of the study. This fact confirms the importance of a SEP to individuals with T1DM, a chronic disease that requires close attention to complex constant care beyond a daily routine. These preliminary results support the application of this methodology in the treatment of individuals with T1DM in the Brazilian context: simple approaches for effective management.
